# Cost-effectiveness of extracorporeal CPR for out-of-hospital cardiac arrest: a trial-based Markov model with a lifetime horizon

**DOI:** 10.1093/ehjacc/zuag058

**Published:** 2026-04-16

**Authors:** Anina F van de Koolwijk, Andrea Gabrio, Thijs S R Delnoij, Martje M Suverein, Stijn E D M Eussen, Brigitte A B Essers, Annemieke Oude Lansink-Hartgring, Renicus C Hermanides, Luuk C Otterspoor, Carlos V Elzo Kraemer, Alexander P J Vlaar, Joris J van der Heijden, Erik Scholten, Corstiaan A den Uil, Dinis Dos Reis Miranda, Sakir Akin, Jesse de Metz, Iwan C C van der Horst, Jos G Maessen, Roberto Lorusso, Marcel C G van de Poll

**Affiliations:** Department of Intensive Care Medicine, Maastricht University Medical Center+, Maastricht University, Maastricht, The Netherlands; Department of Methodology & Statistics, Maastricht University, Maastricht, the Netherlands; Department of Intensive Care Medicine, Maastricht University Medical Center+, Maastricht University, Maastricht, The Netherlands; Department of Cardiology, Maastricht University Medical Centre+, Maastricht University, Maastricht, The Netherlands; Department of Intensive Care Medicine, Maastricht University Medical Center+, Maastricht University, Maastricht, The Netherlands; Department of Intensive Care Medicine, Maastricht University Medical Center+, Maastricht University, Maastricht, The Netherlands; Department of Clinical Epidemiology and Medical Technical Assessment, Maastricht University Medical Center+, Maastricht University, Maastricht, the Netherlands; Department of Critical Care, University Medical Center Groningen, Groningen, the Netherlands; Department of Cardiology, Isala Clinics, Zwolle, the Netherlands; Department of Intensive Care, Catharina Hospital, Eindhoven, the Netherlands; Department of Intensive Care, Leiden University Medical Center, Leiden University, Leiden, the Netherlands; Department of Intensive Care, Amsterdam University Medical Center, University of Amsterdam, Amsterdam, the Netherlands; Department of Intensive Care, University Medical Center Utrecht, Utrecht University, Utrecht, the Netherlands; Department of Intensive Care, St. Antonius Hospital, Nieuwegein, the Netherlands; Department of Intensive Care, Erasmus Medical Center, Erasmus University, Rotterdam, the Netherlands; Department of Cardiology, Erasmus Medical Center, Erasmus University, Rotterdam, the Netherlands; Department of Intensive Care, Maasstad Hospital, Rotterdam, the Netherlands; Department of Intensive Care, Erasmus Medical Center, Erasmus University, Rotterdam, the Netherlands; Department of Intensive Care, Haga Hospital, The Hague, the Netherlands; Department of Intensive Care, OLVG, Amsterdam, the Netherlands; Department of Intensive Care Medicine, Maastricht University Medical Center+, Maastricht University, Maastricht, The Netherlands; Cardiovascular Research Institute Maastricht (CARIM), Maastricht University, Maastricht, the Netherlands; Department of Cardiothoracic Surgery, Maastricht University Medical Center+, Maastricht University, Maastricht, the Netherlands; Department of Cardiothoracic Surgery, Maastricht University Medical Center+, Maastricht University, Maastricht, the Netherlands; Department of Intensive Care Medicine, Maastricht University Medical Center+, Maastricht University, Maastricht, The Netherlands; School for Nutrition and Translational Research in Metabolism (NUTRIM), Maastricht University, Maastricht, the Netherlands

**Keywords:** Out-of-hospital cardiac arrest, Refractory arrest, Extracorporeal cardiopulmonary resuscitation, Cost-effectiveness, Markov model

## Abstract

**Aims:**

Extracorporeal cardiopulmonary resuscitation (ECPR) can restore circulation in refractory out-of-hospital cardiac arrest (OHCA). A trial-based analysis with a 1-year horizon showed limited cost-effectiveness of this demanding procedure. However, arguably, long-term incremental health benefits may justify high initial incremental costs. We assessed the cost-effectiveness of ECPR compared with conventional cardiopulmonary resuscitation (CCPR) for OHCA with a lifetime horizon using trial-based data.

**Methods:**

Healthcare and societal costs and quality adjusted life years (QALY), assessed using EQ-5D-5L, were simulated over a 20-year period following ECPR or CCPR for OHCA using a Markov model. Data from the per-protocol population of a multicentre randomized controlled trial comparing ECPR with CCPR were used as input parameters. The incremental cost-effectiveness ratio (ICER) was expressed as Euros per QALY. Probabilistic and deterministic sensitivity analyses were performed.

**Results:**

We used data from 33 ECPR and 47 CCPR patients. Mean ± SD costs after 1 year were €26.372 ± 28.237 vs. €10.356 ± 37.706, and survival was 15% vs. 9% in patients treated with ECPR vs. CCPR. Over a lifetime horizon, mean incremental costs and QALYs of ECPR were €160.969 and 0.66, respectively, resulting in an ICER of €242.122/QALY. At a willingness-to-pay threshold of €80.000 per QALY gained, the probability of ECPR being cost-effective was 46%. The costs of non-survivors in both arms and the QALYs gained were the major drivers of the ICER.

**Conclusion:**

Extracorporeal cardiopulmonary resuscitation for refractory OHCA has a low probability of being cost-effective. To enhance cost-effectiveness, improving ECPR effectiveness and reducing hospital costs of ECPR non-survivors are mandatory.

## Graphical Abstract

**Graphical Abstract:**
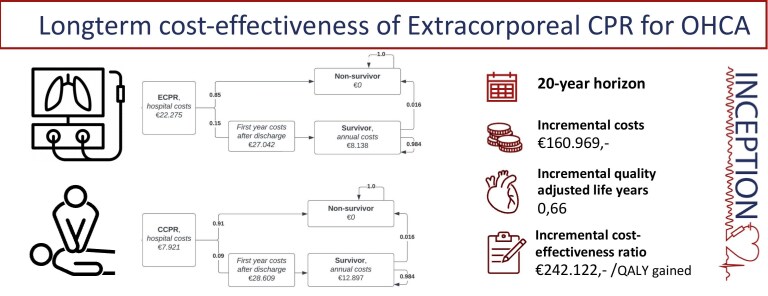

## Introduction

Out-of-hospital cardiac arrest (OHCA) is a life-threatening condition with high mortality varying between 82 and 100%,^1^ especially in refractory arrest, when conventional measures fail to achieve return of spontaneous circulation.^2^ Some patients with a refractory arrest are considered eligible for extracorporeal cardiopulmonary resuscitation (ECPR). Extracorporeal cardiopulmonary resuscitation refers to the use of veno-arterial extracorporeal membrane oxygenation (ECMO) in CPR, which restores circulation and can serve as a bridge to therapy or recovery.

Survival rates up to 40% have been achieved with ECPR for refractory OHCA in well-organized and exceptionally dedicated systems.^3–5^ However, high survival rates are not invariably reproduced outside such centres of excellence,^6–8^ where survival with good neurological outcome ranges between 10% and 20%.^7,9^ The recent multicentre pragmatic INCEPTION trial found a survival rate of 15% after ECPR, compared with 9% in patients treated with conventional CPR (CCPR). Extracorporeal cardiopulmonary resuscitation requires highly skilled personnel and costly resources. The increased use of diagnostic and therapeutic interventions in patients resuscitated through ECPR further drives the cost of ECPR. While the implementation of ECPR increases, it remains unclear if survival and quality of life justify these higher costs.

A cost-effectiveness analysis conducted alongside the INCEPTION trial, which to our knowledge is the only available trial-based cost-effectiveness analysis for ECPR in OHCA, found a low probability of cost-effectiveness after 1 year.^10^ While trial-based cost-effectiveness analyses are grounded on individual patient level and reflect observed outcomes directly from clinical settings, they typically employ a short time horizon and do not consider potential future incremental health benefits that may justify high initial incremental costs. Since ECPR is generally applied in relatively young patients and since many OHCA survivors experience a good health-related quality of life, it is worthwhile to explore the long-term health economic consequences of ECPR for OHCA. Previous publications have used Markov models to estimate long-term cost-effectiveness of ECPR using retrospective data or assumptions as input.^11^

We aimed to investigate the lifetime cost-effectiveness of in-hospital ECPR for OHCA compared with CCPR. To this end we performed a health economic analysis of the INCEPTION trial using a Markov model under a lifetime perspective.

## Methods

### INCEPTION trial background

The INCEPTION trial was a multicentre, randomized controlled trial conducted in the Netherlands, comparing the clinical effectiveness of ECPR and CCPR in patients with refractory OHCA. The trial protocol and primary outcomes have been published previously.^6,12^ The study protocol was approved by the ethics committee of Maastricht University (METC 162039). It was registered at clinicaltrials.gov (NCT03101787). In summary, adult patients with witnessed, refractory OHCA (no ROSC despite 15 min of advanced life support), with an initial shockable rhythm, could be included. Patients were randomized before arrival at the hospital, and if patients assigned to the ECPR group had no ROSC at arrival at the emergency department, ECMO was implemented. Standard post-resuscitation care was delivered according to current guidelines and institutional protocols. A total of 134 patients were included from May 2017 to February 2021.

### Patients

Patients aged 18–70 years with witnessed refractory OHCA due to initial ventricular arrhythmia, where bystander basic life support (BLS) was initiated, could be included. Patients could be excluded based on their medical history (specified in supplements), in case of known contraindications for ECPR, or if the expected time interval was more than 60 min between arrest and initiation of the cannulation procedure. Patients whose actual time interval exceeded 60 min, after randomization, were retained in the study.

For this analysis, we used the per-protocol population, to estimate of cost-effectiveness of actual ECPR delivery in accordance with generally accepted indication criteria.^12^ Most importantly, this excluded patients who regained ROSC before arrival at ED, together with patients for whom ECPR was initiated more than 60 min after the start of the cardiac arrest. All crossovers were also excluded. Patients treated with extracorporeal life support for respiratory or cardiac failure after successful conventional resuscitation were not classified as crossovers but were considered as part of regular post-resuscitation care.

### Economic evaluation

**Figure 1:**
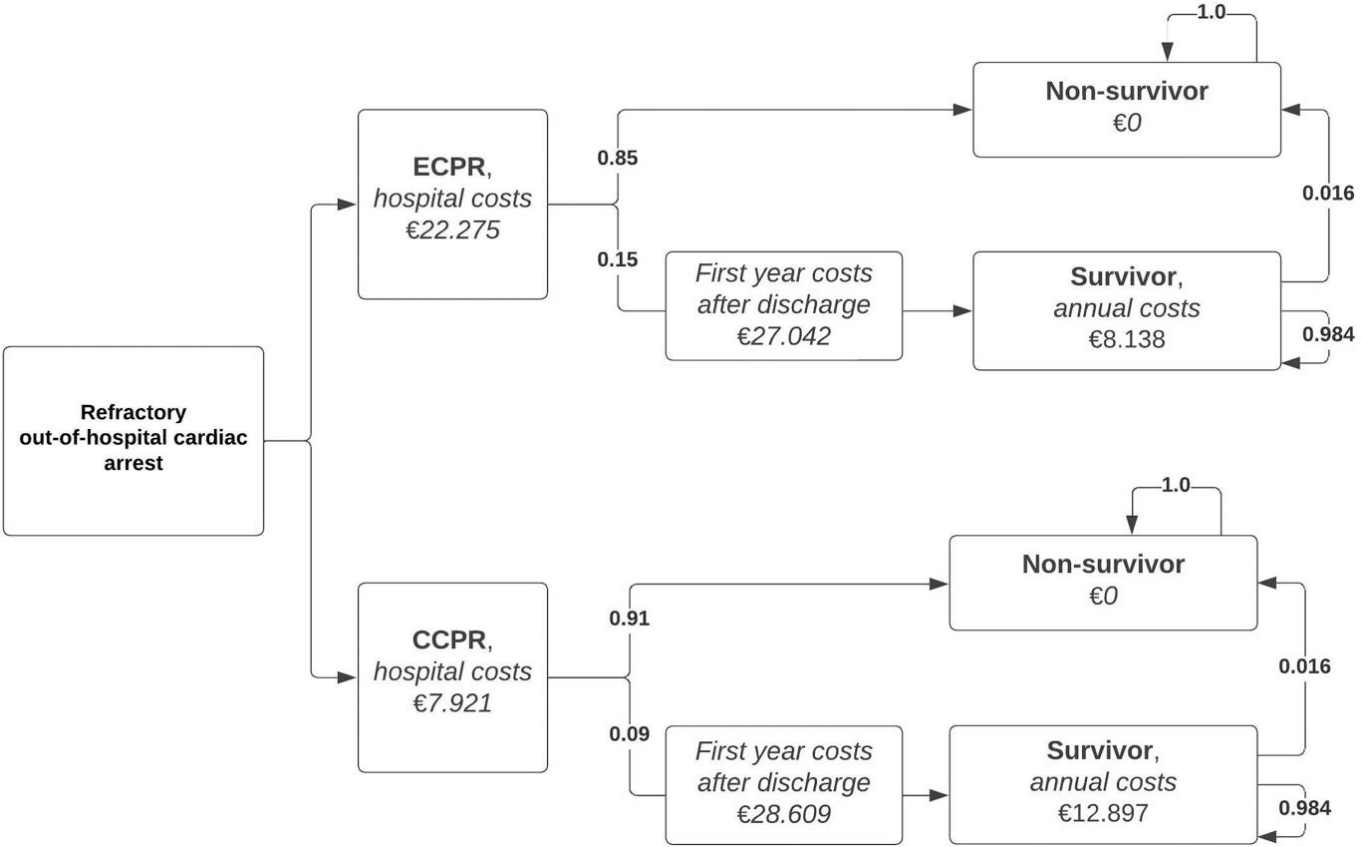
Model diagram Markov model. Costs are derived from actual costs in the per-protocol population of the INCEPTION trial: hospital costs are the mean for all patients (*Table 1*). First-year costs after discharge and annual costs are the mean costs of survivors only (see Supplementary material online, *Table S2*).

A cost-utility analysis was conducted, with results expressed in terms of the incremental cost-effectiveness ratio (ICER), defined as the cost difference divided by the difference in quality adjusted life years (QALYs) between patients treated with ECPR vs. patients treated with CCPR. Prospective costs and QALYs were recorded alongside the trial for a period of one year after randomization, from a societal perspective as described elsewhere.^10^ Trial data were extrapolated beyond the study period to assess cost-effectiveness of ECPR over a lifetime horizon (i.e. 20 years), using a decision-analytic model. Using the statistical software R version 4.5.1^13^ and the Heemod package, we developed a Markov state transition model with two states: ‘survivor’ and ‘non-survivor’ (*Figure 1*). In accordance with Dutch health economic guidelines, we applied an annual discount rate of 3% for costs and an 1.5% annual discount rate for utilities, as well as a willingness-to-pay (WTP) threshold of 80.000 euros per QALY gained.^14^

### Costs

During the trial, all medical costs, including pre-hospital and emergency medical services, emergency department and cannulation costs, intensive care and hospital admission costs, therapeutic and diagnostic interventions, and post-discharge costs, were collected. A detailed description of how costs were calculated was published previously.^10^ In addition, medical and societal costs after discharge were recorded during the follow-up, using the iMTA Medical Consumption Questionnaire (iMCQ) and iMTA Productivity Cost Questionnaire (iPCQ) until 1 year after the arrest. For the annual costs beyond the follow-up period, we used post-discharge costs, without costs for rehabilitation. These annual costs were outpatient visits, re-hospitalizations, ED visits, general practitioner visits, homecare, and medication costs. Mean costs were calculated for surviving ECPR patients, non-surviving ECPR patients, surviving CCPR patients, and non-surviving ECPR patients.

### Health Utility Index

The Health Utility Index (HUI) was determined using the EQ-5D-5L questionnaire,^15^ a validated tool for assessing health-related quality of life and designed for use in health-economic evaluations. This questionnaire records scores in five dimensions: mobility, self-care, usual activities, pain/discomfort, and anxiety. The scores of each domain are converted into a HUI, using county-specific tariffs.^16^ For the Netherlands, this HUI score ranges from −0.45 to 1.0. A score of 1.0 represents perfect health; non-surviving patients are assigned a HUI of 0. The mean HUI after 1 year was used as an input parameter. Since there was no significant difference in HUI between ECPR and CCPR survivors, all patients were assigned the same HUI value of 0.74.^17^ Quality adjusted life years were calculated by multiplying the time of a particular health state by the HUI. For HUI before the OHCA, the mean HUI for 50 years and throughout the general Dutch population was used.^16^

### Survival probabilities

In agreement with results from the per-protocol analysis of the trial, 1-year survival was set at 9% for CCPR and 15% for ECPR.^18^ The annual probability of survival was set at 98.4%, based on data from Statistics Netherlands (CBS).^19^ Research shows that patients who survive 30 days post arrest only have a limited reduction in 10-year survival compared with the general population, especially when the arrest is due to a ventricular arrythmia.^20^

### Sensitivity analyses

A deterministic sensitivity analysis was performed, to examine the impact of specific factors (e.g. costs of non-surviving patients, ED costs, hospital costs, and discharge costs) on the ICER. For all input data, the upper and lower values were used, based on twice the standard deviation.

As part of probabilistic sensitivity analysis, we conducted a Monte Carlo simulation with 5000 iterations, using cost and QALY parameters defined by observed means from the trial, standard deviations, and the number of patients per group. Results in terms of mean incremental estimates were plotted on the cost-effectiveness plane,^21^ and the probability that ECPR is cost-effective compared with CCPR across varying WTP thresholds was visually represented through the cost-effectiveness acceptability curve.^22^

To assess the long-term cost-effectiveness of pragmatic ECPR delivery within a broader perspective of OHCA care, we performed a secondary scenario analysis using the intention-to-treat data of the INCPETION trial. Additionally, to test the impact of potential diminished long-term survival probabilities of OHCA survivors, we also performed sensitivity analyses using different annual mortality probabilities based on literature.^20,23^

### Cost-effectiveness in relation to ECPR survival rates

To investigate the potential effect of an increased clinical effectiveness of ECPR on the ICER, we repeated the Markov model while varying the input value for ECPR survival up to 40%, which is approximately the highest ECPR survival rate reported by centres of excellence.^4^ Other input parameters, including the survival rate with CCPR, were kept unchanged. Resulting ICERs were plotted against crude ECPR survival. As this was already an exploratory analysis, no further sensitivity analyses were performed.

## Results

Of the 134 patients who enrolled in the trial, 53 were excluded from the per-protocol analysis based on prespecified criteria. The flow of patients from the intention-to-treat to the per-protocol population and their outcomes have been described extensively elsewhere.^18^ Briefly, of the remaining 80 patients, 33 were treated with ECPR and 47 with CCPR. Almost all patients were male (91%), with a mean age of 56 ± 9 years. All patients had a shockable rhythm as initial rhythm. Thirty-day survival was 5/33 (15%) in ECPR patients and 5/47 (9%) in CCPR patients (adjusted OR 1.9; 95% CI −0.4–9.3; *P*-value 0.39). All baseline characteristics are shown in the supplemental appendix (see Supplementary material online, *Table S1*).

### Cost analysis

The costs of ECPR and CCPR patients are shown in *Table 1*. Mean hospital costs for ECPR vs. CCPR patients were €22.275 ± 23.876 and €7.921 ± 30.631, respectively. Of the costs in the ECPR group, €6.586 ± 0 was for ECMO cannulation. Total costs in the first year were €26.372 ± 28.237 for ECPR patients and €10.356 ± 37.706 for CCPR patients (mean difference €16.016). Apart from the costs for the placement and continuation of ECMO, the higher costs in the ECPR group were driven by a longer hospital admission (+€5.559), more blood transfusions (+€1.192), and (invasive) diagnostic and therapeutic procedures such as computer tomography, coronary angiography, and percutaneous coronary interventions (+€2.196). Post-discharge costs were €4.097 ± 12.338 for ECPR patients and €10.356 ± 37.706 for CCPR patients. Costs per survival status are specified in the supplemental appendix (see Supplementary material online, *Table S2*); the mean costs for an ECPR non-survivor were €18.994 ± 23.107 vs. €1.924 ± 4.595 for a CCPR non-survivor.

**Table 1:** Costs based upon INCEPTION trial, per protocol data

	ECPR (N = 33)		CCPR (N = 47)	
	Mean cost ± SD	Mean resource use	Mean cost ± SD	Mean resource use
*Hospital costs*	*€22.275 ± 23.876*		*€7.921 ± 30.631*	
Pre-hospital costs	€964 ± 0	1	€964 ± 0	1
ECMO implantation	€6.586 ± 0	1	€0 ± 0	0
ECMO days	€828 ± 665	2.37	€0 ± 0	0
Nursing days	€11.001 ± 18.599	0.73	€5.442 ± 24.392	0.12
Medication	€324 ± 588	—	€58 ± 225	—
Transfusion	€1.480 ± 3.086	0.55	€288 ± 1.944	0.04
Diagnostic interventions				
CAG	€495 ± 355	0.66	€142 ± 295	0.12
Other	€181 ± 222	—	€13 ± 57	—
Therapeutic interventions	€1.934 ± 3.545	—	€968 ± 3.840	—
PCI	€997 ± 982	0.52	€288 ± 670	0.15
*Post-discharge costs*	*€4.097 ± 12.338*		*€2.424 ± 10.472*	
General practitioner	€11 ± 34	0.15	€13 ± 70	0.06
Homecare formal/informal	€301 ± 1.614	—	€140 ± 927	—
Medication	€47 ± 166	—	€166 ± 907	—
ED visit/EMS	€26 ± 110	0.06	€30 ± 171	0.04
Outpatient clinic visits	€27 ± 88	—	€17 ± 67	—
Hospital stay	€144 ± 829	0.03	€0	0.02
Rehabilitation facility stay	€0	0	€149	0.04
Productivity loss	€1.872 ± 6.635	—	€1.059 ± 5.191	—
*Total costs first year*	*€26.372 ± 28.237*		*€10.356 ± 37.706*	
*Annual costs*	*€8.138 ± 10.305*		*€12.897 ± 14.476*	
*Health Utility Index before OHCA* ^16^	0.86		0.86	
*Health Utility Index after OHCA* ^17^	0.74		0.74	

x categories without reported mean resource use contain multiple different resources with different unit costs compile

### Incremental cost-effectiveness ratio

Using these input parameters, the mean incremental cost-effectiveness over 1 year was calculated at €360.720/QALY (€16.016 cost difference/6% survival difference × 0.74 healthy utility). The Markov model showed that over a period of 20 years, the mean incremental costs in patients treated with ECPR in comparison with CCPR for OHCA were calculated to be €160.969, while the mean increment in QALYs in ECPR-treated patients was calculated to be 0.66, resulting in an ICER of €242.122 per QALY gained.

### Sensitivity analysis

**Figure 2:**
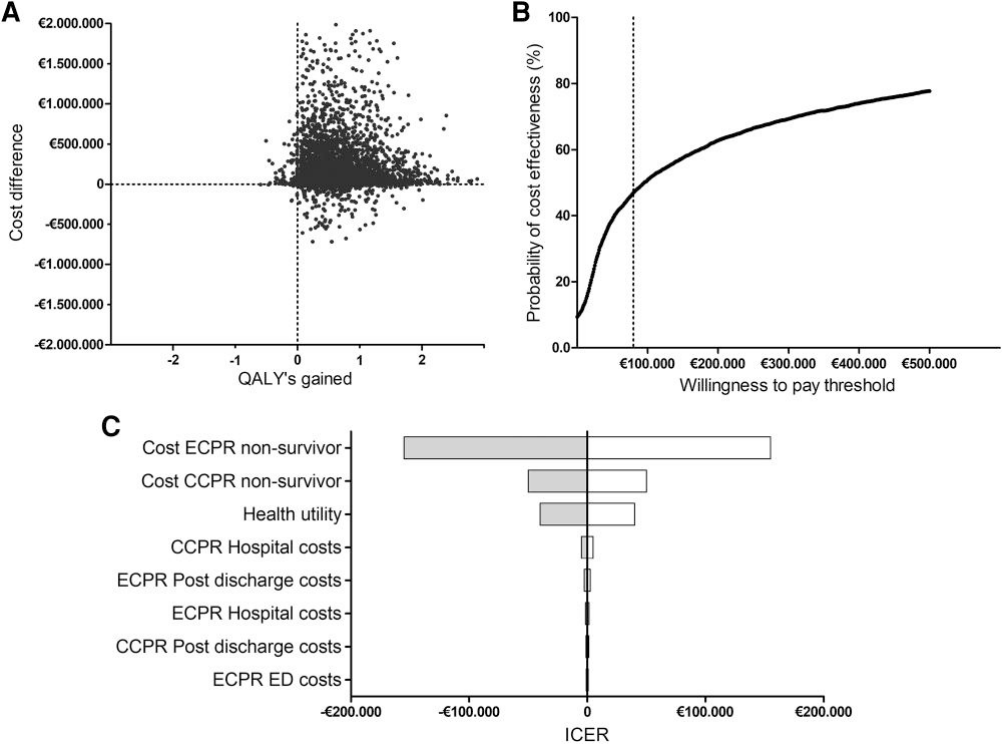
(*A*) Cost-effectiveness plane of ECPR compared with CCPR. (*B*) Probability of cost-effectiveness with different willingness-to-pay thresholds. (*C*) Different factors influencing the incremental cost-effectiveness ratio (ICER). ECPR, extracorporeal cardiopulmonary resuscitation. CCPR, conventional cardiopulmonary resuscitation. ED, emergency department. ICER, incremental cost-effectiveness ratio. QALY, quality adjusted life years.

The probabilistic sensitivity analysis showed that, in this cost-effectiveness analysis with a 20-year time horizon after the arrest of the per-protocol population in the INCEPTION trial, 88% of all ratios are located in the quadrant where ECPR is more effective and more costly than CCPR (*Figure 2A*). The acceptability curve shows that at a WTP threshold of 80.000 euros, the probability of ECPR being cost-effective is 46% (*Figure 2B*). The deterministic sensitivity analysis showed that the most important factors influencing the ICER are the difference in hospital costs between ECPR and CCPR non-survivors, and the difference in QALYs between both groups, which is entirely dependent on the survival rate of ECPR, as we applied a similar utility score for both groups (*Figure 2C*).

Intention-to-treat analysis showed a cost difference of €65.056 and a mean increment in QALYs of 0.44, resulting in an ICER of €146.781. Costs and input data are specified in Supplementary material online, *Table S3*. With increased annual mortality probability, costs increased, and effect difference decreased, resulting in a higher ICER (see Supplementary material online, *Table S4*).

### Association between cost-effectiveness and incremental effectiveness

**Figure 3:**
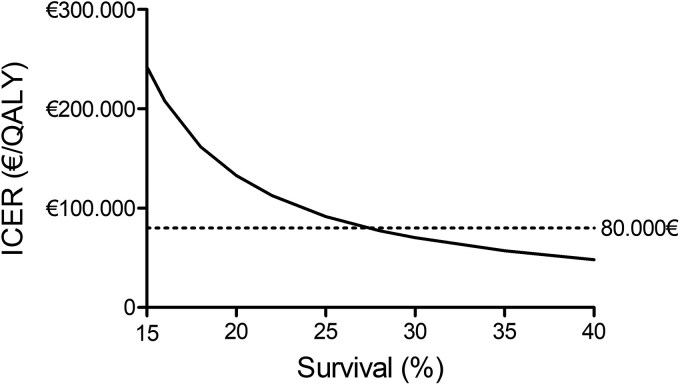
Incremental cost-effectiveness ratio (ICER) per percentage of survival. ICER, incremental cost-effectiveness ratio. QALY, quality adjusted life years.

Repeating the Markov model with varying ECPR survival rates (while keeping all other parameters unchanged) showed that the lifetime ICER improved with an increasing ECPR survival. At an ECPR survival rate of 27%, a WTP threshold of €80.000 per QALY was approached (*Figure 3*). In this hypothetical model, the ICER was €48.085 per QALY with an ECPR survival of 40%.

## Discussion

In this paper, we presented a long-term cost-effectiveness analysis of in-hospital ECPR for OHCA based on data from the INCEPTION trial. To our knowledge, this study is the first cost-effectiveness analysis on ECPR for OHCA with a lifetime horizon that is based on actual trial data. In the base-case analysis, compared with CCPR, we found a lifetime ICER for ECPR of €242.122 QALY gained.^10^ At a WTP threshold of €80.000, the probability of ECPR being cost-effective was 46%. This considerable decision uncertainty is primarily due to the large standard deviations in both costs and utility in OHCA survivors. Our results do not clearly favour either strategy from a cost-effectiveness perspective, and the decision to implement ECPR should therefore be seen in the context of risk tolerance of a healthcare system. Policymakers should weigh the risk of avoiding potentially preventable deaths to the probability of ineffective healthcare spending. From our results, it can be deduced that the probability of the latter is greater than the probability of the first, when ECPR is applied in a pragmatic way as was done during the INCEPTION trial. Notably any investment beyond pragmatic implementation, e.g. dedicated team readiness, regular training, and simulation programmes or pre-hospital strategies, will increase costs and warrant a further increase in incremental effectiveness to meet the WTP threshold. The limited cost-effectiveness is primarily determined by the high costs associated with non-surviving ECPR patients compared with CCPR patients, as well as the low incremental effectiveness of ECPR compared with CCPR. Interestingly, these costs are only partly affected by the direct ECMO costs and are almost equally determined by the costs of ICU and hospital admission and by diagnostic and therapeutic procedures.

The 1-year cost-effectiveness calculated by our model, an ICER of €360.720/QALY, differs from the ICER of the per-protocol population in our previous cost-effectiveness analysis that was fully based on the trial data (259.068€/QALY).^10^ The more favourable ICER in the previous trial analysis can be ascribed to the between-group difference in the EQ-5D-5L health utility score more in favour of ECPR survivors that was found in the INCEPTION trial (0.73 vs. 0.84).^17^ However, since this difference was not significant, and data from other groups also provide no indication that HRQoL of ECPR survivors is actually better than that of CCPR survivors,^24,25^ we decided against introducing a different health utility for CCPR and ECPR survivors in our model.

In current study we chose a predefined, per-protocol approach to estimate the long-term cost-effectiveness among patients who actually received the intervention according to the study protocol and according to generally accepted indication criteria to estimate a ‘maximum cost-effectiveness’ of pragmatic ECPR implementation to the right patients. However, this approach may not fully capture the broader cost-effectiveness of running a pragmatic ECPR-ready programme within an existing healthcare system. Such a programme is inevitably accompanied by false call-outs due to patients achieving ROSC before cannulation and by patients that are cannulated despite not meeting strict criteria. A sensitivity analysis of the intention-to-treat population showed that the high proportion of false call-outs reduces the average costs per patient treated within an ECPR-ready programme but also the proportion of patients actually receiving ECPR, troubling the assessment of ECPR cost-effectiveness.

While trial-based cost-effectiveness analyses reflect observed outcomes directly from clinical settings, they typically use a short time horizon. In situations where a substantial long-term clinical benefit is to be expected, the use of a short time horizon may lead to an underestimation of cost-effectiveness. The increase in the ICER value obtained from using a lifetime horizon (€242.122/QALY) compared with that from a 1-year analysis (€360.720/QALY) supports the idea that future health gains play an important role in determining the cost-effectiveness of ECPR. However, we noted that the ICER value estimated from the analysis using a lifetime horizon still exceeds the WTP threshold that is applied by most European healthcare systems.^26^

Different model-based studies have suggested a more favourable cost-effectiveness ratio for ECPR.^27–29^ The differences in results may be explained by several factors. Results of model-based analyses should be interpreted cautiously, as assumptions drive the results. For example, in the study of Bharmal et al. and Dennis et al., the assumption was made that survival in patients treated with CCPR was 0%. Doan et al. used a theoretical study cohort with a neurological favourable survival of 1.9% in the CCPR group. The per-protocol analysis of the INCEPTION trial and a post-hoc analysis of the Prague OHCA trial^6,30^ have clearly shown that survival through CCPR is not negligible in patients who would qualify as ECPR candidates. Underestimating CCPR survival may overestimate the incremental clinical effectiveness and thereby the cost-effectiveness of ECPR. Other studies^31^ did not include costs for non-surviving patients, while we demonstrated that the cost difference between ECPR and CCPR is primarily driven by non-surviving patients. Unlike previous research, we included costs for loss of productivity, as patients eligible for ECPR are generally younger individuals who still participate in the working society.

The INCEPTION trial has been criticized for its pragmatic approach and resulting practice variation, which may limit its validity compared with studies in dedicated centres. However, low flow times, as marker of system performance, align with typical outcomes in uncontrolled settings.^32^ The incremental favourable outcome of ECPR over CCPR in our analysis matches the findings of a recent meta-analysis.^33^ Taken together, the literature supports the external validity and representatives of the INCEPTION trial and hence the current analysis.^8,33^

Considering all associated costs as well as CCPR survival, we calculated that in a healthcare system similar to the one in the Netherlands, an ECPR survival rate of approximately 27% is required to reach a ‘break-even point’ with an ICER of €80.000. It is important to note that additional investments aimed at improving system performance and ECPR survival, such as simulation training, enhanced readiness, and communication infrastructure, should be factored into the total costs of ECPR. These additional expenses further increase the survival rate required for ECPR to become cost-effective.^34^

It has been repeatedly shown that shorter low flow times are associated with greater ECPR survival, suggesting that reducing low flow times may be worth pursuing to improve cost-effectiveness. However, reducing cannulation time also increases the likelihood of cannulating patients who might have achieved ROSC and survival with CCPR. As a result, the incremental effectiveness may not improve proportionality with improved survival rates.^34^

Another approach to improve cost-effectiveness is to reduce the overall costs of the procedure. In our analysis, the ICER was primarily driven by costs associated with non-surviving patients, mainly because of high nursing expenses during ICU and hospital admission, as well as costly procedures. Identifying patients for whom treatment is likely to be futile at an early stage, such as through early neuroprognostication, could help to reduce the costs of a prolonged, futile ICU and hospital stay.

### Strengths and limitations

The main strength of this study is the use of input data that were directly derived from trial-based evidence collected in a multicentre trial, ensuring a high level of internal and external validity. The use of real-world data enhances the reliability of the cost estimates and HRQoL and improves the applicability of the findings. Additionally, the study focuses on long-term cost-effectiveness (i.e. over a lifetime of 20 years), providing valuable insights beyond short-term outcomes.

A limitation of the current study is the need to rely on modelling assumptions for data beyond the first year. Due to the limited number of surviving patients, the costs associated with survivors were based on a small sample size and showed considerable variation between individuals. Since we aimed to inform our model inputs by truly comparative data, we deliberately minimized post-hoc adjustments to the dataset. We did however equalize health utility scores amongst groups as there is no evidence that ECPR leads to a different functional outcome in OHCA survivors than CCPR. Also we used a single health state in survivors, which resulted in a fairly simple Markov model. This is in in line with the very low proportion of unfavourable neurology in survivors in our dataset. This approach however may not be applicable for other healthcare systems where withdrawal of life sustaining treatment is less common when neurological prognosis is poor. The varying degree of neurological outcome in OHCA survivors is however captured by implementing the standard deviation derived from our study cohort in the deterministic and probabilistic sensitivity analyses. Annual healthcare costs after the first year were derived from healthcare costs during the first year, excluding rehabilitation stay which we assumed would not carry over to further years. To account for possible further functional improvement and reduction of attributable costs beyond the first year, we applied an annual discount rate according to guidelines for health economic analyses. Finally, the possible health benefits associated with an increased number of organ donations were not considered in the current study. Evidence shows that the implementation of ECPR leads to a threefold increase in the number of patients who are eligible for brain–death organ donation compared with CCPR.^35^ It may be argued that these health benefits should be considered when assessing the cost-effectiveness of ECPR. However, these potential benefits have not been incorporated into the current analysis, as no organ donations were performed within the trial-based data.

## Conclusion

This model-based cost-effectiveness analysis, using trial-based input parameters, investigated the cost-effectiveness of in-hospital ECPR for refractory OHCA with a lifetime horizon and found an ICER of €242.122 per QALY gained for ECPR. When using a WTP of €80.000, we found a probability of 46% that ECPR was cost-effective compared with CCPR. To enhance cost-effectiveness, improving ECPR effectiveness and reducing hospital costs for ECPR non-survivors are mandatory.

## Supplementary Material

zuag058_Supplementary_Data

## Data Availability

Pseudonymized data are available from corresponding author upon request.
