# Supplementary material for: Cost-effectiveness of extracorporeal CPR for out-of-hospital cardiac arrest: a trial-based Markov model with a lifetime horizon
Source: Eur Heart J Acute Cardiovasc Care. 2026 Apr 16;15(7):516–23. doi: 10.1093/ehjacc/zuag058 (PMC13344173; doi:10.1093/ehjacc/zuag058)
Supplement: zuag058_Supplementary_Data [file zuag058_supplementary_data.docx]

Supplements

# INCEPTION Investigators

| Martje M. Suverein, M.D. | Department of Intensive Care, Maastricht University Medical Center, Maastricht, the Netherlands |
| --- | --- |
| Thijs S.R. Delnoij, M.D. | Department of Intensive Care, Maastricht University Medical Center, Maastricht, the Netherlands |
| Roberto Lorusso, M.D., Ph.D. | Department of Cardiothoracic Surgery, Maastricht University Medical Center, Maastricht, the Netherlands |
| George J. Brandon Bravo Bruinsma, M.D., Ph.D. | Department of Cardiothoracic Surgery, Isala Klinieken, Zwolle, the Netherlands |
| Luuk Otterspoor, M.D., Ph.D. | Department of Intensive Care, Catharina Hospital, Eindhoven, the Netherlands |
| Carlos V. Elzo Kraemer, M.D., . | Department of Intensive Care, Leiden University Medical Center, Leiden, The Netherlands |
| Alexander P.J. Vlaar, M.D., Ph.D. | Department of Intensive Care, Amsterdam University Medical Center location AMC, Amsterdam, the Netherlands |
| Joris J. van der Heijden, M.D. | Department of Intensive Care, University Medical Center Utrecht, Utrecht, The Netherlands |
| Erik Scholten, M.D. | Department of Intensive Care, St. Antonius Hospital, Nieuwegein, The Netherlands |
| Corstiaan den Uil, M.D., Ph.D. | Department of Intensive Care, Erasmus Medical Center, Rotterdam, The Netherlands |
| Tim Jansen, M.D., Ph.D. | Department of Intensive Care, HagaZiekenhuis, Den Hague, The Netherlands |
| Bas van den Bogaard, M.D., Ph.D. | Department of Intensive Care, OLVG, Amsterdam, The Netherlands |
| Marijn Kuijpers, M.D. | Department of Intensive Care, Isala Klinieken, Zwolle, the Netherlands |
| Ka Yan Lam, M.D. | Department of Cardiothoracic Surgery, Catharina Hospital, Eindhoven, the Netherlands |
| José M. Montero Cabezas, M.D.Ph.D. | Department of Cardiology, Leiden University Medical Center, Leiden, The Netherlands |
| Antoine H.G. Driessen, M.D., Ph.D. | Department of Cardiothoracic Surgery, Amsterdam University Medical Center location AMC, Amsterdam, The Netherlands |
| Saskia Z.H. Rittersma, M.D., Ph.D. | Department of Cardiology, University Medical Center Utrecht, Utrecht, The Netherlands |
| Bram G. Heijnen, M.D. | Department of Intensive Care, St. Antonius Hospital, Nieuwegein, The Netherlands |
| Dinis Dos Reis Miranda, M.D., Ph.D. | Department of Intensive Care, Erasmus Medical Center, Rotterdam, The Netherlands |
| Gabe Bleeker, M.D., Ph.D. | Department of Cardiology, HagaZiekenhuis, Den Hague, The Netherlands |
| Jesse de Metz, M.D., Ph.D. | Department of Intensive Care, OLVG, Amsterdam, The Netherlands |
| Renicus S. Hermanides, M.D., Ph.D. | Department of Cardiology, Isala Klinieken, Zwolle, the Netherlands |
| Jorge Lopez Matta, M.D. | Department of Intensive Care, Leiden University Medical Center, Leiden, The Netherlands |
| Susanne Eberl, M.D., Ph.D. | Department of Anesthesia, Amsterdam University Medical Center location AMC, Amsterdam, The Netherlands |
| Dirk W. Donker, M.D., Ph.D. | Cardiovascular and Respiratory Physiology, TechMed Center, University of Twente, Enschede, The Netherlands  Department of Intensive Care, University Medical Center Utrecht, Utrecht, The Netherlands |
| Robert J. van Thiel, M.D. | Department of Intensive Care, Erasmus Medical Center, Rotterdam, The Netherlands |
| Sakir Akin, M.D., Ph.D. | Department of Intensive Care, HagaZiekenhuis, Den Hague, The Netherlands |
| Oene van Meer, M.D. | Department of Emergency Medicine, Leiden University Medical Center, Leiden, The Netherlands |
| José Henriques, M.D., Ph.D. | Department of Cardiology, Amsterdam University Medical Center location AMC, Amsterdam, The Netherlands |
| Karen C. Bokhoven, M.D. | Department of Intensive Care, Erasmus Medical Center, Rotterdam, The Netherlands |
| Henrik Endeman, M.D., Ph.D. | Department of Intensive Care, Erasmus Medical Center, Rotterdam, The Netherlands |
| Jeroen J.H. Bunge, M.D. | Department of Intensive Care, Erasmus Medical Center, Rotterdam, The Netherlands  Department of cardiology, Thorax Center, Erasmus University Medical Center Rotterdam, The Netherlands |
| Martine E. Bol, | Department of Intensive Care, Maastricht University Medical Center, Maastricht, the Netherlands |
| Bjorn Winkens, Ph.D. | Department of Methodology & Statistics, Maastricht University, Maastricht, The Netherlands |
| Brigitte Essers, Ph.D. | Department of Clinical Epidemiology and Medical Technical Assessment, Maastricht University Medical Center, Maastricht, the Netherlands |
| Patrick W. Weerwind, CCP, Ph.D. | Department of Cardiothoracic Surgery, Maastricht University Medical Center, Maastricht, the Netherlands |
| Jos G. Maessen, M.D., Ph.D. | Department of Cardiothoracic Surgery, Maastricht University Medical Center, Maastricht, the Netherlands |
| Marcel C.G. van de Poll, M.D., Ph.D. | Department of Intensive Care, Maastricht University Medical Center, Maastricht, the Netherlands |

# Dutch ECLS study group

Department of Critical Care, University Medical Center Groningen, Groningen, the Netherlands

- Dr. Joep M. Droogh

Department of Intensive Care, Amsterdam University Medical Center, Amsterdam, the Netherlands

- Dr. Ing. Charissa E. van den Brom

Department of Critical Care, University Medical Center Utrecht, Utrecht, the Netherlands

- Dr. Jeannine A. J. Hermens

Adult Intensive Care Unit, Leiden University Medical Center, Leiden, the Netherlands

- Drs. Carlos V. Elzo Kraemer

- Drs. Jeroen A. Janson

- Drs. Jorge Lopez Matta

Department of Intensive Care, Maastricht University Medical Center, Maastricht, the Netherlands

- Dr. Marcel C.G. van de Poll

Department of Critical Care, OLVG, Amsterdam, the Netherlands

- Dr. Bas van den Bogaard

Department of Critical Care, Catharina Hospital, Eindhoven, the Netherlands

- Dr. Luuk Otterspoor

Department of Intensive Care, St. Antonius Hospital, Nieuwegein, the Netherlands

- Mw. Ineke van de Pol

Department of Intensive Care, Isala Klinieken, Zwolle, the Netherlands

- Drs. Laurien van Koppenhagen

Adult Intensive Care Unit, Erasmus Medical Center, Rotterdam, the Netherlands

- Dr. Christiaan L. Meuwese

- Drs. Jeroen J.H. Bunge

**In- and exclusion criteria INCEPTION-trial [13]**

| Inclusion criteria | Exclusion criteria (if known before randomization) |
| --- | --- |
| 1. ≥18 - ≤70 years 2. Witnessed OHCA 3. Initial rhythm of VF/VT or AED-shock administered 4. Bystander BLS 5. No ROSC within 15 minutes | 1. ROSC with sustained hemodynamic recovery within 15 minutes 2. Terminal heart failure (NYHA III or IV) 3. Severe pulmonary disease (COPD GIII of GIV) 4. Oncological disease 5. Pregnancy 6. Bilateral femoral vessel bypass surgery 7. Pre-arrest CPC score of 3 or 4 8. Multiple trauma (Injury Severity Score> 15) 9. Advance health care directive 10. Expected initiation of cannulation >60 min after arrest |

*OHCA: out-of-hospital cardiac arrest, VF: ventricular fibrillation, VT: ventricular tachycardia, AED: automatic external defibrillator, BLS: basic life support, ROSC: return of spontaneous circulation, CPC: cerebral performance category*

**Table S1. Baseline characteristics, per protocol (1)**

|  | **ECPR (N = 33)** | **CCPR (N = 47)** |
| --- | --- | --- |
| Age – years ±SD | 55 ± 11 | 56 ± 8 |
| Male – no. (%) | 30 (91) | 43 (92) |
| Primary shockable rhythm – no. (%) | 33 (100) | 47 (100) |
| Cause of arrest – no. (%)  Acute myocardial infarction  Secondary arrhythmia  Dissection  Pulmonary embolus  Metabolic or electrolyte  Neurologic  Intoxication  Other / unkown | 25 (76)  3 (9)  2 (6)  1 (3)  1 (3)  0 (0)  1 (0)  2 (6) | 38 (81)  7 (15)  0 (0)  0 (0)  0 (0)  0 (0)  0 (0)  3 (6) |
|  |  |  |
| Medical history – no. (%)  Acute coronary syndrome  Coronary artery disease  PCI  CABG  Chronic heart failure  Cerebral vascular accident  Peripheral artery disease  Diabetes mellitus  Hypertension  Hypercholesterolemia  Current smoker | 5 (15)  4 (14)  2 (6)  1 (3)  1 (3)  2 (6)  0 (0)  4 (12)  11 (33)  5 (15)  11 (33) | 8 (17)  6 (13)  5 (11)  2 (4)  1 (2)  7 (15)  3 (6)  4 (9)  9 (19)  11 (23)  11 (23) |
| Duration of arrest – min ±SD | 66 ± 18 | 58 ± 18 |
| Hospitalization – days [IQR] | 1 [1-8] | 7 [0-22] |
| ICU stay – days [IQR] | 1 [1-5] | 1 [1-7] |
| Survived to ICU discharge – no. (%) | 5 (15) | 5 (11) |
| Survived to hospital discharge – no. (%) | 5 (15) | 4 (9) |

*ECPR: extracorporeal cardiopulmonary resuscitation, CCPR: conventional cardiopulmonary resuscitation, PCI: percutaneous coronary intervention, CABG: coronary artery bypass grafting, ICU: intensive care unit*

**Table S2. Costs per survival group, per protocol**

|  | **ECPR survivor (n=5)** | **ECPR non-survivor (n= 28)** | **CCPR survivor (n=4)** | **CCPR non-survivor (n=43)** |
| --- | --- | --- | --- | --- |
| *Hospital costs (mean ± SD*)  Pre-hospital costs  ECMO implantation  ECMO days  Nursing days  Medication  Transfusion  Diagnostic interventions  CAG  Other  Therapeutic interventions  PCI | *€40.646 ± 21.439*  €964 ± 0  €6.586 ± 0  €1.048 ± 494  €26.305 ± 15.607  €418 ± 329  €708 ± 547  €594 ± 332  €210 ± 242  €5.464 ± 8.554  €1.161 ± 1.060 | *€18.994 ± 23.107*  €964 ± 0  €6.586 ± 0  €790 ± 692  €8.269 ± 17.966  €307 ± 626  €1.618 ± 3.333  €477 ± 362  €176 ± 222  €1.304 ± 1.156  €1.232 ± 985 | *€72.390 ± 89.634*  €964 ± 0  €0 ± 0  €0 ± 0  €56.758 ± 71.136  €499 ± 641  €3.332 ± 6.665  €743 ± 0  €122 ± 163  €9.447 ± 10.808  €1.452 ± 968 | *€1.942 ± 4.595*  €964 ± 0  €0 ± 0  €0 ± 0  €668 ± 4.006  €17 ± 79  €5 ± 36  €86 ± 240  €3 ± 122  €180 ± 568  €180 ± 568 |
| *Post discharge costs (mean ±SD*)  General practitioner  Homecare formal/informal  Medication  ED visit / EMS  Outpatient clinic visits  Hospital stay  Rehabilitation facility stay  Productivity loss | *€27.042* ± 21.029  €72 ± 63  €1.986 ± 4.084  €313 ± 343  €171 ± 256  €7.068 ± 15.804  €953 ± 2.131  €0 ± 0  €11.835 ± 14.294 | *€0 ± 0* | *€28.609 ± 26.136*  €155 ± 217  €1.653 ± 3.139  €1.958 ± 2.821  €358 ± 542  €3.052 ± 6.105  €0 ± 0  €1.757 ±2.079  €10.901 ± 14.764 | *€0 ± 0* |
| *Total costs first year (mean ±SD)* | *€67.689 ± 16.111* | *€18.994 ± 23.107* | *€101.000 ± 97.624* | *€1.924 ± 4.595* |
| *Annual costs after first year (mean ±*SD) | *€8.138 ± 10.305* | *€0 ± 0* | *€12.897 ± 14.476* | *€0 ± 0* |
| *Health utility index before OHCA* | 0.86 | | 0.86 | |
| *Health utility index after OHCA* | 0.74 ± 0.27 | 0 ± 0 | 0.74 ± 0.27 | 0 ± 0 |
| *Probabilty of survival (%)* | 15 | | 9 | |
| *Probability of annual mortality* | 0.016 | 0 | 0.016 | 0 |

*ECMO: extracorporeal membrane oxygenation, CAG: coronary angiography, PCI**: percutaneous coronary intervention, ED: emergency department, EMS: emergency medical service, OHCA: out-of-hospital cardiac arrest*

**Table S3. Costs per survival group, intention to treat analysis**

|  | **ECPR survivor (n=14)** | **ECPR non-survivor (n= 56)** | **CCPR survivor (n=11)** | **CCPR non-survivor (n=51)** |
| --- | --- | --- | --- | --- |
| *Hospital costs (mean ± SD*)  Pre-hospital costs  ECMO implantation  ECMO days  Nursing days  Medication  Transfusion  Diagnostic interventions  CAG  Other  Therapeutic interventions  PCI | *€31.380* ± 16.168  €964 ± 0  €2.715 ± 3.788  €399 ± 610  €19.889 ± 12.349  €310 ± 424  €430 ± 749  €583 ± 316  €224 ± 208  €6.165 ± 6.031  €893 ± 1.004 | *€15.064* ± 17.361  €964 ± 0  €5.124 ±2.658  €517 ±592  €5.596 ± 13.186  €248 ± 487  €1.048 ± 2.607  €530 ± 338  €169 ± 224  €1.227 ± 1.123  €1.204 ± 949 | *€49.694* ±55.152  €964 ± 0  €0 ± 0  €0 ± 0  €36.401 ±45.067  €364 ±442  €1.211 ± 4.019  €743 ± 0  €75 ± 117  €9.647 ± 7.999  €1.056 ±1.011 | *€3.811* ± 8.747  €964 ± 0  €392 ±1.566  €68 ± 261  €1.979 ± 6.964  €65 ±235  €9 ± 46  €131 ± 286  €11 ± 38  €265 ±672  €265 ± 672 |
| *Post discharge costs (mean ±SD*)  General practitioner  Homecare formal/informal  Medication  ED visit / EMS  Outpatient clinic visits  Hospital stay  Rehabilitation facility stay  Productivity loss | *€20.457*±18.503  €54 ± 51  €738 ± 2.464  €360 ± 348  €122 ± 216  €258 ± 283  €411 ± 1.280  €0 ± 0  €12.798 ± 13.539 | *€0 ± 0* | *€28.916*±22.660  €195 ± 189  €601 ± 1.910  €1.118 ± 1.744  €208 ± 364  € 292 ± 213  €1.250 ±3.207  €3.817 ± 9.036  €12.352 ± 13.537 | *€0 ± 0* |
| *Total costs first year (mean ±SD)* | *€51.838*±22.726 | *€15.064*±17.361 | *€78.611*±65.317 | *€3.811* ± 8.747 |
| *Annual costs after first year (mean ±*SD) | *€4.658* ±6.567 | *€0 ± 0* | *€14.610*±12.711 | *€0 ± 0* |
| *Health utility index before OHCA* | 0.86 | | 0.86 | |
| *Health utility index after OHCA* | 0.74 ± 0.27 | 0 ± 0 | 0.74 ± 0.27 | 0 ± 0 |
| *Probabilty of survival (%)* | 20 | | 16 | |
| *Probability of annual mortality* | 0.016 | 0 | 0.016 | 0 |

*ECMO: extracorporeal membrane oxygenation, CAG: coronary angiography, PCI: percutaneous coronary intervention, ED: emergency department, EMS: emergency medical service, OHCA: out-of-hospital cardiac arrest*

**Table S4. Additional sensitivity analyses**

|  | Cost difference | Effect difference | ICER |
| --- | --- | --- | --- |
| Annual mortality 1.75%* | €161.136 | 0.66 | 245.101 |
| Annual mortality 3.2%** | €162.630 | 0.59 | 274.867 |
| Annual mortality 6% (2) | €164.962 | 0.49 | 336.922 |
| Intention to treat population, annual mortality 1.6% | €65.056 | 0.44 | 146.781 |

*10% increased mortality compared to general population

** Twice increased mortality compare to general population (CBS, Netherlands (3)

1. Ubben JFH, Suverein MM, Delnoij TSR, Heuts S, Winkens B, Gabrio A, et al. Early extracorporeal CPR for refractory out-of-hospital cardiac arrest - A pre-planned per-protocol analysis of the INCEPTION-trial. Resuscitation. 2024;194:110033.

2. Andrew E, Nehme Z, Wolfe R, Bernard S, Smith K. Long-term survival following out-of-hospital cardiac arrest. Heart. 2017;103(14):1104-10.

3. Levensverwachting; geslacht, leeftijd (per jaar en periode van vijf jaren). In: Statistiek CBvd, editor. 2024.
